# Clinical Results of Diffractive, Refractive, Hybrid Multifocal, and Monofocal Intraocular Lenses

**DOI:** 10.1155/2018/8285637

**Published:** 2018-06-25

**Authors:** Agnieszka Dyrda, Ana Martínez-Palmer, Daniel Martín-Moral, Amanda Rey, Antonio Morilla, Miguel Castilla-Martí, Janny Aronés-Santivañez

**Affiliations:** ^1^Department of Ophthalmology, Hospital Universitario del Mar and Hospital de la Esperanza, Pompeu and Fabra University, Barcelona, Spain; ^2^Institut Català de Retina, Barcelona, Spain; ^3^Valles Ophthalmology Research, Hospital General de Catalunya, Barcelona, Spain

## Abstract

**Purpose:**

To present the outcomes of hybrid multifocal and monofocal intraocular lenses (IOLs) and to compare with refractive and diffractive multifocal IOLs (MFIOLs).

**Methods:**

Three hundred twenty eyes (160 patients) underwent cataract surgery with randomized IOLs bilateral implantation. Changes in uncorrected and distance-corrected logMAR distance, intermediate and near (UNVA and DCNVA) visual acuity (VA), contrast sensitivity (CS), presence of dysphotopsia, spectacle independence, and patient satisfaction were analyzed.

**Results:**

Postoperative VA in the hybrid (OptiVis) group was improved in all distances (*p* < 0.001). OptiVis acted superiorly to monofocal IOLs in UNVA and DCNVA (*p* < 0.001 for both) and to refractive ones in DCNVA (*p* < 0.005). Distance, mesopic, without glare CS in OptiVis was lower than in the monofocal group and similar to other MFIOLs. No differences in dysphotopsia pre- and postoperatively and spectacle independence in near for OptiVis and refractive MFIOLs were detected. OptiVis patients were more satisfied than those with monofocal IOLs (*p*=0.015).

**Conclusions:**

After cataract surgery, patients with OptiVis improved VA in all distances. Near and intermediate VA was better than monofocal, and DCNVA was better than the refractive group. CS was lower in OptiVis than in the monofocal group, but there was no difference between MFIOLs. Patient satisfaction was higher in OptiVis than in the monofocal group. This trial is registered with NCT03512626.

## 1. Introduction

Nowadays, cataract surgery is a refractive procedure. Although monofocal intraocular lenses (IOLs) ensure excellent distance acuity, patients require spectacles for near and intermediate vision [1]. Multifocal IOLs (MFIOLs) have different depth of focus capabilities within the optical zone and effectively achieve good visual acuity (VA) for far and near distances, guaranteeing spectacle independence. MFIOLs use a refractive, a diffractive, or a combination of both designs. One of the main disadvantages of refractive multifocal IOLs is their pupil dependence, while the loss of energy is the main drawback of the diffractive design. Studies showed that MFIOLs had increased dysphotopsia and decreased contrast sensitivity (CS) compared with monofocal IOLs [1, 2]. These side effects can limit visual function and reduce patient's quality of life [3]. Comparison of aspheric and spherical IOLs showed superior visual performance of aspheric IOLs, especially in CS [4, 5]. The OptiVis™ MFIOL (Aaren Scientific, Inc., Ontario, CA, USA) offers several advantages, as it is a real multifocal hybrid design. The lens is distance dominant and has a central progressive refractive zone within 1.5 mm surrounded by a diffractive zone from 1.5 mm to 3.8 mm of diameter that allows far and near vision in a full range of pupil sizes. The progressive power refractive zone allows far and intermediate vision, and the apodized diffractive design minimizes light loss outside and reduces halos in the far focus. Additionally, aspheric lens periphery improves image contrast in large pupils for different corneal asphericities [6]. Binocular implantation of MFIOLs is preferred to monocular implantation [7].

The purpose of this study was to compare the visual outcomes after cataract surgery with bilateral implantation of a hybrid (refractive-diffractive) multifocal IOL (OptiVis, Aaren Scientific) and a monofocal IOL (AR40e, AMO) and to compare with our previous study of refractive and diffractive multifocal IOLs.

## 2. Patients and Methods

This prospective, randomized, controlled study was conducted at the Ophthalmology Department of the Hospital de la Esperanza, Barcelona, Spain. Institutional review board approval was obtained, and the study adhered to the Declaration of Helsinki. Written informed consent was obtained from all patients. Eligibility was determined based on a complete ophthalmologic examination. Inclusion criteria were senile cataract with Snellen VA ≤ 0.5 and motivation for spectacle independence for near vision. As the study was conducted in the Spanish public health care system, entering the study was the only option to get multifocal lens, as they are not provided by public health care, and the patients were conscious of the possibility of randomization to the monofocal group. Exclusion criteria were corneal astigmatism ≥1.10 diopters (D), irregular astigmatism, axial length <21.5 or ≥25 mm, pupillary diameter in mesopic conditions in distance vision ≤2.5 mm and ≥6 mm, age ≥80 years, ocular pathology that could affect the visual function and/or IOL centering, and intraoperative or postoperative complications. Highly demanding patients and those whose profession could be affected by a multifocal design (professional drivers, jewelers, etc.) were also excluded, as in the Spanish public health care system's secondary procedures needed to satisfy patients' expectation, such as LRIs, LASIK, and PRKs, are not available. Patients were randomly assigned to have bilateral implantation with either a monofocal IOL (AR40e, AMO-Abbott 30 Laboratories Inc., Abbott Park, Illinois, USA) or multifocal IOL (OptiVis, Aaren Scientific, Inc., Ontario, CA, USA).

We used the previously unpublished results of a randomized, controlled study, performed in the same center with the same protocol and methodology, to compare performance of a refractive-diffractive multifocal IOL (OptiVis, Aaren Scientific, Inc., Ontario, CA, USA) with refractive (M-Flex, Rayner Intraocular Lenses Limited, Hove, UK; ReZoom, AMO-Abbott 30 Laboratories Inc., Abbott Park, Illinois, USA) and diffractive (ReSTOR +4, Alcon Laboratories, Inc., Fort Worth, USA) IOLs. Since OptiVis is a hybrid multifocal lens, it is assumed to offer the advantages of both designs.

### 2.1. Preoperative Assessment

Preoperatively, all patients had a full ophthalmologic examination including uncorrected distance visual acuity (UDVA), corrected distance visual acuity (CDVA) at 6 m, uncorrected intermediate visual acuity (UIVA), distance-corrected intermediate visual acuity (DCIVA) at 60 cm (in the OptiVis group only, as they were supposed to provide intermediate distance vision in contrary to the other studied lenses), uncorrected near visual acuity (UNVA), distance-corrected near visual acuity (DCNVA) at 33 cm (all measured using Snellen acuity charts under photopic conditions), refraction, slit lamp biomicroscopy, Goldmann applanation tonometry, and fundoscopy. Monocular and binocular CS were measured in mesopic conditions, without glare at spatial frequencies of 1.5, 3, 6, 12, and 18 cycles per degree (cpd) using the functional acuity contrast test (FACT, OPTEC 6500®, Stereo Optical Co. Inc.). Pupil diameter in distance vision was evaluated using a “Rosenbaum pocket-card.” Spectacle dependence, determined by questionnaire (Do you wear glasses for distance/near vision?), and presence of dysphotopsia (halos, glare), spontaneously mentioned or elicited in response to questioning were also assessed preoperatively. The IOL power was calculated using the SRK/T with an A-constant of 118.4 for AR40e and 118.1 for OptiVis using partial coherence interferometry (IOLMaster 500, Carl Zeiss Meditec AG). Postoperative target refraction was emmetropia.  Table 1 shows the patient demographics.

### 2.2. Intraocular Lenses

The IOLs used in our study are presented in Table 2.

### 2.3. Surgical Technique

The same experienced surgeon (AMP) performed all the surgeries under topical anesthesia using a standard phacoemulsification procedure with Infiniti Vision System (all from Alcon Laboratories, Inc., Fort Worth, TX) and with IOL implantation in the capsular bag through a 2.75 mm clear corneal incision. The incision was performed in the steepest meridian. Both eyes were operated on within 1–4 weeks.

### 2.4. Postoperative Examination

**Figure 1:**
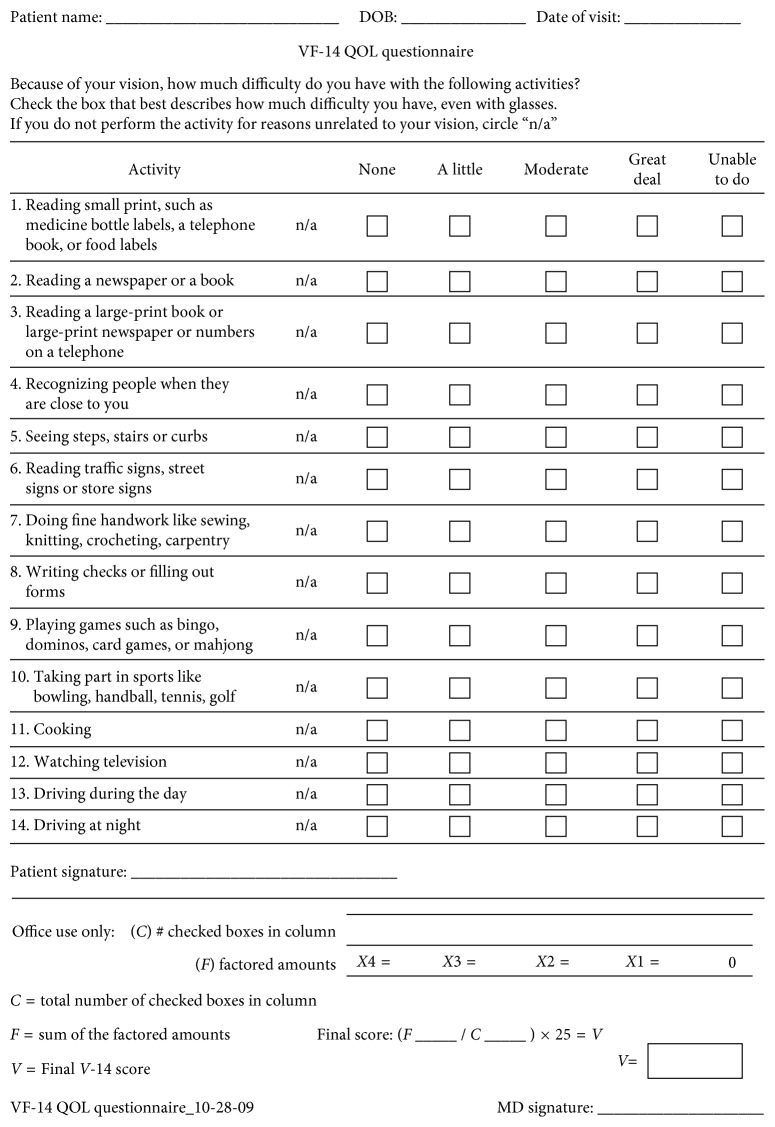


Routine postoperative examinations were performed 1 day, 1 month, and 3 months after surgery. The main and secondary outcomes were assessed at the last follow-up visit, and included UDVA, CDVA, UIVA, DCIVA, UNVA and DCNVA, refraction, CS, pupil diameter, spectacle dependence, and presence of dysphotopsia, as described in the preoperative examination. Patient satisfaction was also assessed with the VF-14 test, consisting of 14 questions evaluating various patient activities (Figure 1). The validity and reproducibility of this test have already been reported [8, 9].

### 2.5. Statistical Analysis

#### 2.5.1. Sample Size

Sixty-four eyes (32 patients) were required per group to detect a statistically significant difference of at least 0.15 in VA between the two groups with statistical power of 80% and an alpha error of 0.05.

Patients were assigned randomly to the multifocal or monofocal group using a 1 : 1 block randomization scheme.

All data were collected in an Excel database (Office 2010, Microsoft Corporation), and statistical analyses were per-formed using SPSS for Windows software (version 22, SPSS Inc., Chicago, IL).

Normality of all data was evaluated using the Kolmogorov–Smirnov test. When parametric analysis was not possible, the differences between preoperative and postoperative data were evaluated with the Mann–Whitney *U* test. The test was also used for comparison of OptiVis with other types of IOL individually for all the parameters except age and CS, which were compared with ANOVA post hoc. The Kruskal–Wallis test was used to detect differences among all groups.

The results are presented as linear diagrams, where the medians are connected and the standard deviation (SD) of each median is presented as a vertical line, and by box plot diagrams, where the bottom and top of the box correspond to the first and third quartiles, and the band inside the box corresponds to the second quartile (the median); the point outside the box is the value between 1.5 and 3 box lengths, while the asterisk represents a value greater than 3 lengths.

Demographic data were used to check whether the preoperative characteristics of the groups differed statistically. The results are expressed as mean ± SD. For all statistical tests, a *p* value of less than 0.05 was considered as statistically significant.

## 3. Results

Each IOL group comprised 64 eyes of 32 patients. All patients completed the 3-month follow-up. No eye was excluded from analysis because of intraoperative or postoperative complications. Although significant differences between OptiVis and AR40e were observed, we assumed that this was aleatory, as it was a randomized clinical trial (Table 1). There was no significant difference in any parameter, except initial CDVA and DCNVA between groups of previously studied MFIOLs (M-Flex, ReZoom, and ReSTOR) and OptiVis (Table 1), so comparison was possible.

### 3.1. Visual Acuity and Refraction

Postoperative VA improved after implantation of OptiVis and AR40e IOLs. Significant differences were found when postoperative and preoperative results were compared for all distance and near uncorrected and corrected VA in the OptiVis group (*p* < 0.001), while this difference was not observed in UNVA in the AR40e group (*p*=0.321). When postoperative results were compared between these studied groups, differences were detected for all VA, except distance VA, as seen in Table 3.

**Figure 2:**
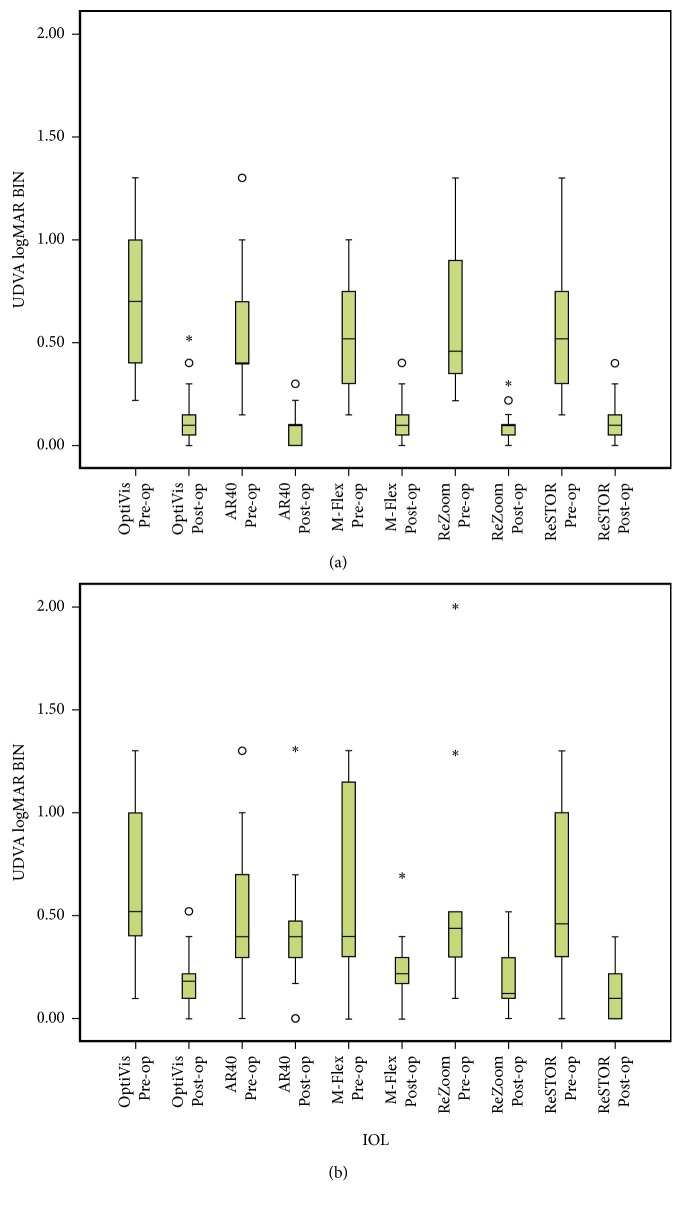
Pre- and postoperative visual performance of (a) UDVA and (b) UNVA of all five IOLs. UDVA BIN: binocular uncorrected distance visual acuity; UNVA BIN: binocular uncorrected near visual acuity; IOL: intraocular lens.

VA was contrasted between all MFIOLs at the final visit and presented in Table 3. No differences in UDVA and CDVA were noticed. Diffractive IOL performed significantly better than OptiVis in UNVA (*p* < 0.009), but this difference became insignificant in DCNVA. While in DCNVA, OptiVis acted significantly better than M-Flex and ReZoom (*p* < 0.001, 0.004, resp.), as noted in Table 3.  Figure 2 shows pre- and postoperative visual performance (UDVA and UNVA) of all five IOLs.

**Figure 3:**
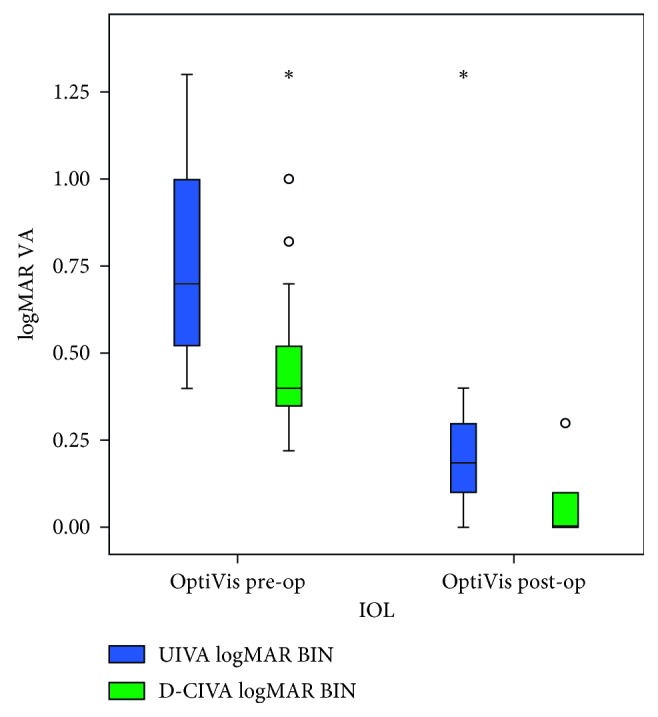
Binocular UIVA and DCIVA pre- and postoperative results in the OptiVis group. UIVA BIN: binocular uncorrected intermediate visual acuity; DCIVA BIN: binocular distance-corrected intermediate visual acuity; IOL: intraocular lens.

As OptiVis was supposed to provide good intermediate VA, we checked the outcomes in this group. The preoperative mean ± SD logMAR UIVA and DCIVA were 0.8 ± 0.33 and 0.23 ± 0.22, respectively, and postoperatively were 0.54 ± 0.31 and 0.04  ±  0.06, respectively. As shown in Figure 3, postoperative UIVA and DCIVA gain were significant (*p* < 0.001 for both).

The predictability of the refractive outcome was good with postoperative mean ± SD spherical equivalent (SE) of 0.17 ± 0.58 and SE within ±0.50 D of the attempted spherical correction in 26 eyes (80%) and within ±1.00 D in 30 eyes (94%) in the OptiVis group. SE was slightly hyperopic in the OptiVis and ReSTOR groups and slightly myopic in the M-Flex and ReZoom groups (Table 3).

### 3.2. Contrast Sensitivity

**Figure 4:**
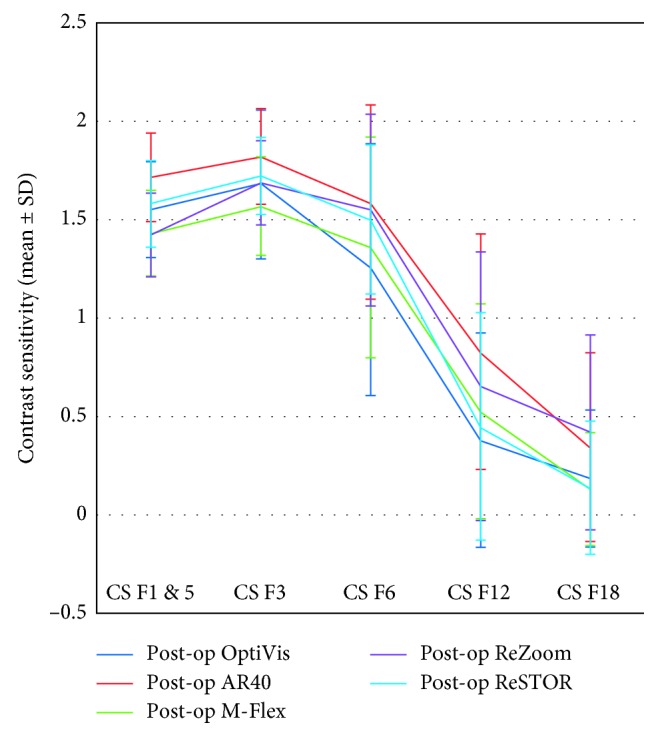
Postoperative mesopic log contrast sensitivity function at far distances without glare in all five IOLs. IOL: intraocular lens; SD: standard deviation; CS: contrast sensitivity; spatial frequencies of 1.5, 3, 6, 12, and 18 cycles per degree.

After cataract surgery in both studied IOLs, multifocal (OptiVis) and monofocal (AR40e), CS at all frequencies: 1.5, 3, 6, 12, and 18 cpd, improved significantly (*p* < 0.001). Under mesopic conditions without glare, distance CS with the multifocal IOL was significantly lower than with the monofocal IOL at any tested frequencies (1.5 cpd, *p* < 0.001; 3 cpd, *p*=0.004; 6 cpd, *p*=0.022; 12 cpd, *p*=0.012; and 18 cpd, *p*=0.017, Kruskal–Wallis), as seen in Figure 4. There was no significant difference between MFIOLs performance after surgery, as presented in Table 4.

### 3.3. Spectacle Independence Evaluation, Dysphotopic Phenomena, and Visual Function

The participants used spectacles less often after surgery (*p* < 0.001); 16% and 9% of patients declared spectacle independence for far distance in the OptiVis and AR40e groups (*p*=0.436), and 50% and 13% for near distance, respectively, with significantly less spectacle dependence for near in the OptiVis group (*p*=0.001). In general, there were no differences in spectacle independence between MFIOLs at tested distances, except for ReZoom at far distance (*p*=0.021) and ReSTOR at near distance (*p*=0.004) when compared with OptiVis (Table 5).

There were no differences in dysphotopsia spontaneously mentioned in the pre- and postoperative assessment (*p*=0.796) or in the questionnaire (*p*=0.802) in the OptiVis group. There were also no differences between MFIOLs (Table 5).

**Figure 5:**
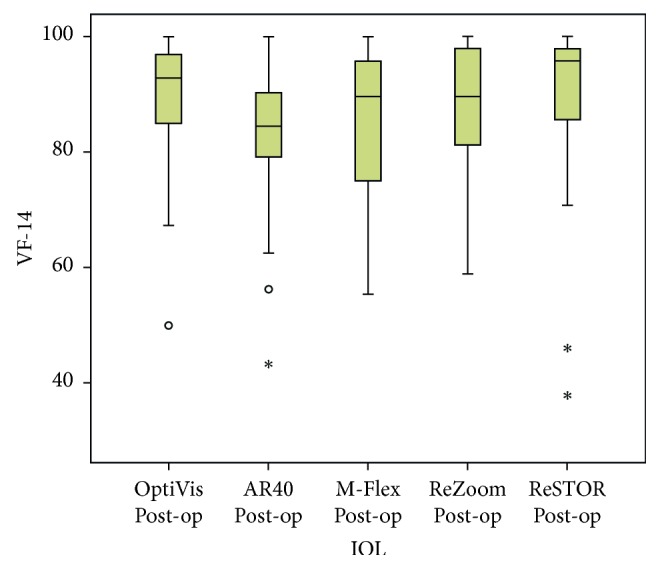
Visual function (VF-14) results in multifocal intraocular lens. VF-14: visual function test; IOL: intraocular lens.

Visual function evaluation by VF-14 questionnaire showed that patients with bilateral OptiVis implantation were more satisfied than monofocal users (*p*=0.015). As shown in Figure 5, patients' satisfaction was high after the multifocal procedure. OptiVis and ReSTOR had the highest scores in the VF-14 survey: 89.28 ± 11.11 and 89.51 ± 14.85, respectively, but the results were not statistically different from other MFIOLs (Table 5).

## 4. Discussion

MFIOLs provide spectacle independence after cataract surgery. The classic design (refractive or diffractive) allows bifocality with good visual function at distance and near but with poor intermediate vision. More recent models were designed to have lower near addition to improve intermediate vision. However, these IOLs still provide only average visual results for intermediate distances or improve intermediate vision at the expense of near VA [10], so better solutions are sought. A new idea was to fuse two classical designs in one MFIOL. OptiVis, a hybrid MFIOL, currently unique on the market to our knowledge, has three different zones: (1) a progressive power refractive zone within central diameter of 1.5 mm that allows far and intermediate vision, (2) a diffractive apodized bifocal zone with a diameter of 1.5–3.8 mm that allows far and near vision for a full range of pupil sizes and less halos, and (3) aspheric distance periphery to improve CS. We compared the visual performance of OptiVis to monofocal IOL (AR40e) in a clinical setting. We also compared the results of this clinical trial with our previously unpublished study, as we considered it interesting to assess the superiority of a hybrid model over refractive (M-Flex, Rayner, ReZoom, and AMO) and diffractive (ReSTOR and Alcon) MFIOLs. As we know, ReZoom and ReSTOR have been for years the reference in refractive and diffractive design, with which the new multifocal lens models were usually compared.

As expected, there were no significant differences in UDVA and CDVA between all studied MFIOLs and monofocal IOLs (*p*=0.131, Kruskal–Wallis), but MFIOLs performed much better in UNVA and DCNVA (*p* < 0.001 for both, Kruskal–Wallis). Randomized, controlled trials (RCTs) [11–15] and meta-analyses of RCTs [1, 16, 17] comparing the results of multifocal and monofocal IOLs concluded that uncorrected near vision is improved by implantation of a multifocal IOL, resulting in lower spectacle dependence for near tasks without compromising distance VA [18, 19], as shown in our study: OptiVis patients were less spectacle dependent for near vision than AR40e patients (*p*=0.001). No statistical differences were found in distance VA between different MFIOLs [20], as in our study.

After 3-month follow-up, VA of 0.3 logMAR in UDVA, UIVA, and UNVA was achieved by 93.75%, 93.75%, and 81.25% of OptiVis patients, respectively, compared to 3-month outcomes after bilateral OptiVis implantation in the study by Piovella and Bosc [6] 96.8%, 71.3%, and 92.6%, respectively. The difference between our study and Piovella's [6] in UIVA and UNVA might be caused by a choice of distinct measures in intermediate (60 cm versus 70 cm, resp.) and near distance (33 cm versus 40 cm, resp.). The choice of 60 and 33 cm was dictated by distance measurements in our previously conducted study in order to be able to compare OptiVis with refractive and diffractive IOLs as we wanted to assess the superiority of the hybrid model. Moreover, as far as we know, 60 and 33 cm are used frequently in literature in order to check visual outcomes. On the contrary, we could not expect that other studies using OptiVis with perhaps other distances would not be published.

Bilateral OptiVis implantation after cataract extraction provided useful UIVA and DCIVA to our patients. Mean logMAR VA in DCIVA was much higher in our study (0.04 ± 0.06) than in the spherical diffractive (0.38 ± 0.14) and aspheric diffractive MFIOLs (0.14 ± 0.17) at the 60 cm distance [10]. Binocular UIVA was significantly better in the refractive MFIOLs than in the diffractive MFIOLs [21] and was similar to OptiVis results. In the study by Chiam et al. [22], UIVA was 0.24 ± 0.1 in the ReZoom group, similar to the results obtained for the OptiVis group (0.23 ± 0.22).

MFIOLs, as we know, provide good vision in wide range of distances, but intermediate vision might be insufficient for daily life. That is why MFIOL design is currently evolving. Progress towards trifocal IOLs with useful third focus for intermediate vision is a good example. According to many studies, trifocal IOLs improved intermediate vision when compared with bifocal IOLs, without impairing distance and near vision [23–25], but another study reported that bifocal IOLs provide similar UIVA [26]. To our knowledge, there are only 2 systematic reviews and meta-analysis published [27, 28]. Unfortunately, none included OptiVis. In both studies, the quality of the evidence in terms of intermediate VA was very low, as there was a limited number of studies [24, 26, 29] included, and heterogeneity was high. Mean UIVA in the trifocal group was insignificantly better than that in the bifocal group, but when analyzing the defocus curves, trifocal IOLs had significantly better performance [27, 28]. The mean UIVA in the trifocal group was 0.33 ± 0.10 (70 cm, Finevision Micro F, PhysIOL S.A.) [26], 0.06 ± 0.07 (66 cm, AT LISA Tri 839 MP, Carl Zeiss Meditec, Dublin, CA) [29], and 0.07 ± 0.05 (66 cm, Finevision Micro F) [24]. As seen, our results (0.23 ± 0.22) were better than the ones of Jonker et al. [26] but clearly worse than two other studies included in the meta-analysis [24, 29]. Although the meta-analysis did not support superiority of trifocal IOLs in intermediate VA, there are increasing number of studies providing excellent results of trifocal IOLs as the one of Bilbao-Calabuig et al. [30] where binocular mean UIVA measured at 80 cm in 4282 eyes (AT LISA Tri 839 MP) and 5802 eyes (Finevision Micro F) was −0.05 ± 0.14 and −0.05 ± 0.12, respectively.

Extended depth of focus (EDOF) IOLs are the latest variation. Tecnis Symfony IOL (Abbott Medical Optics, Inc.) differs from multifocal IOLs, as it provides a continuous range of vision by spreading out light along a range, instead of splitting it between two distinct points. By minimizing chromatic aberration, the lens is maximizing image quality and contrast. Its weakness is suboptimal VA in near distance. Although there are still only few studies published, EDOF IOLs provided successful visual restoration after cataract surgery with excellent visual outcomes across all distances [31]. UIVA mean values are similar to or better than those obtained for different types of multifocal IOLs, including diffractive bifocal and trifocal IOLs [11, 24, 32–36]. Cochener et al. [31] noted mean binocular UIVA of 0.13 ± 0.16 (70 cm), while Pedrotti et al. [36] noted mean binocular UIVA of 0.10 ± 0.09 (60 cm), much better than intermediate VA outcomes in OptiVis patients. In Pedrotti's study [36], UIVA of 20/32 (in Snellen) was reached by 100% of patients with Tecnis Symfony IOL (60 cm), whereas only by 40.6% of our OptiVis (60 cm) and 44.3% of Piovella's patients (70 cm) [6].

The four MFIOLs studied (OptiVis, M-Flex, ReZoom, and ReSTOR) were compared for near vision. OptiVis and ReSTOR had better DCNVA than refractive models, but OptiVis performed worse than diffractive MFIOL in UNVA and equal to refractive MFIOLs. Our results in UNVA are similar to those presented by Piovella and Bosc [6]. Cumulative UNVA of 20/25 or better (in Snellen) was achieved by 37.4% of our patients and by 40.4% of Piovella's patients [6]. Diffractive IOL performed better in UNVA, as it has higher addition (+4.0 D) [37]. Moreover, in the hybrid lens, intermediate focus is potentiated at the expense of near focus. This was reflected in significantly less spectacle dependence for near vision in ReSTOR, but such strong addition impaired intermediate VA and led to a really short reading distance [38]. This was the reason for lowering addition to +3 D in a newer model of ReSTOR. Moreover, slightly hyperopic postoperative SE was observed in the OptiVis group, and this could also partially prejudice the UNVA (Table 3).

Despite the benefits of uncorrected VA at various distances, MFIOLs are associated with certain disadvantages. Firstly, they provide lower CS when compared with monofocal IOLs [11, 13, 39], especially in mesopic conditions [40], as confirmed by our findings. Although CS in individuals with multifocal IOLs is diminished, it is generally within the normal range of contrast in age-matched phakic individuals [41]. Patients in our study did not have a reduction in CS after implantation of the OptiVis. Moreover, they improved significantly (*p* < 0.001) in low cpd and gained significantly (*p*=0.003) in the high frequencies (12 and 18 cpd) due to cataract surgery. These results were comparable to a previously published report by Hohberger et al. [42], who evaluated CS in normal subjects in a similar age cohort. Our results could not be compared to those reported by Piovella and Bosc [6], as they studied CS in photopic and scotopic conditions after glare. Although diffractive MFIOLs cause light energy dispersion among the secondary orders of diffraction, they appear to be comparable to refractive multifocal IOLs in terms of CS [43, 44], as seen in our study.

Secondly, halos and glare are more often reported with a multifocal IOL than with a monofocal lens [1]. Nevertheless, OptiVis patients did not complain, as no differences were observed in the incidence of dysphotopsia before and after surgery (*p*=0.796 for dysphotopsia spontaneously mentioned and *p*=0.802 when asked). Fifty-nine percent of OptiVis patients reported dysphotopsia postoperatively, similar to the 54% of patients in the study by Piovella and Bosc [6] We did not observe differences between MFIOL groups (Kruskal–Wallis test: *p*=0.458 for dysphotopsias spontaneously mentioned and *p*=0.254 when asked), although the literature suggests that refractive MFIOLs are associated with more dysphotopic phenomena than diffractive MFIOLs [11]. However, Cochener et al. [2] did not find any significant differences in the incidence of halos with different types of multifocal IOLs, which is consistent with our findings.

Patients demonstrate high satisfaction with bilateral cataract surgery. According to a meta-analysis by de Silva et al. [1], there were no significant differences in visual function in far distance reported by patients with multifocal and monofocal implantation, but MFIOLs obtained a better score in visual function when evaluating tasks at near distance [11, 13, 14]. Satisfaction was higher in the OptiVis group than in the AR40e group (*p*=0.015). VF-14 mean ± SD score was 89.3 ± 11.1 in OptiVis patients, comparable to the 89.5 ± 12.6 found in the study by Nijkamp et al. [14]. Patients' comfort with MFIOLs was high and similar to previously published studies [1].

In summary, patients with bilateral implantation of OptiVis were satisfied, although uncorrected intermediate and near VA were not optimal. As mentioned, intermediate VA of our patients with OptiVis was similar to the previously published results with refractive MFIOLs and better than ReSTOR outcomes but worse than with trifocal and EDOF IOLs. DCNVA was better than with refractive MFIOLs, but UNVA was slightly worse than with diffractive MFIOL.

Although the idea seemed good in principle, VA should be better. Consequently, the emergence of trifocal IOLs and the search for new accommodative solutions are justified by the need to improve the quality of vision at all distances.

## Figures and Tables

**Table 1:** Patient demographics and clinical information.

Parameter	Group 1 OptiVis	Group 2 AR40e	Group 3 M-Flex	Group 4 ReZoom	Group 5 ReSTOR	*p* value between groups
Number of patients	32	32	32	32	32	
Number of eyes	64	64	64	64	64	

Age (y)						1 versus 2 0.004^†^
Mean ± SD	67.0 ± 4.9	72.31 ± 3.26	70.3 ± 5.0	68.2 ± 6.1	69.2 ± 6.9	1 versus 3 0.184^†^
						1 versus 4 0.929^†^
Range	55; 74	63; 77	57; 76	52; 78	49; 77	1 versus 5 0.593^†^

Sex (F)						1 versus 2 0.296^*∗*^
Percentage	72%	59%	56%	66%	56%	1 versus 3 0.196^*∗*^
						1 versus 4 0.593^*∗*^
						1 versus 5 0.196^*∗*^

UDVA (logMAR)						1 versus 2 0.029^*∗*^
Mean ± SD	0.75 ± 0.36	0.55 ± 0.30	0.56 ± 0.25	0.61 ± 0.33	0.52 ± 0.28	1 versus 3 0.051^*∗*^
						1 versus 4 0.124^*∗*^
Range	1.30; 0.22	1.30; 0.15	1.00; 0.15	1.30; 0.22	1.30; 0.15	1 versus 5 0.072^*∗*^

CDVA (logMAR)						1 versus 2 0.003^*∗*^
Mean ± SD	0.39 ± 0.21	0.24 ± 0.11	0.26 ± 0.11	0.21 ± 0.10	0.24 ± 0.12	1 versus 3 0.020^*∗*^
						1 versus 4 0.000^*∗*^
Range	1.00; 0.15	0.52; 0.05	0.52; 0.10	0.52; 0.05	0.52; 0.05	1 versus 5 0.003^*∗*^

UNVA (logMAR)						1 versus 2 0.030^*∗*^
Mean ± SD	0.67 ± 0.36	0.50 ± 0.34	0.64 ± 0.44	0.58 ± 0.47	0.59 ± 0.41	1 versus 3 0.395^*∗*^
						1 versus 4 0.058^*∗*^
Range	1.30; 0.10	1.30; 0.00	1.30; 0.00	2.00; 0.10	1.30; 0.00	1 versus 5 0.204^*∗*^

DCNVA (logMAR)						1 versus 2 0.000^*∗*^

Mean ± SD	0.49 ± 0.15	0.15 ± 0.12	0.17 ± 0.14	0.14 ± 0.11	0.16 ± 0.12	1 versus 3 0.000^*∗*^
						1 versus 4 0.000^*∗*^
Range	0.80; 0.10	0.40; 0.00	0.52; 0.00	0.30; 0.00	0.40; 0.00	1 versus 5 0.000^*∗*^

SE (D) RE						1 versus 2 0.876^*∗*^
Mean ± SD	−0.74 ± 2.66	−0.77 ± 2.12	−0.09 ± 1.77	−0.41 ± 2.50	0.20 ± 1.92	1 versus 3 0.427^*∗*^
						1 versus 4 0.604^*∗*^
Range	−6.00; 3.75	−4.75; 3.75	−4.50: 3.75	−5.50; 3.00	−4.00; 3.25	1 versus 5 0.189^*∗*^

SE (D) LE						1 versus 2 0.755^*∗*^
Mean ± SD	−0.50 ± 2.65	−0.20 ± 2.04	0.03 ± 1.94	−0.44 ± 2.70	−0.02 ± 2.58	1 versus 3 0.406^*∗*^
						1 versus 4 0.859^*∗*^
Range	−6.00; 4.00	−5.25; 3.5	−4.75; 3.75	−5.75; 3.75	−10.00; 3.75	1 versus 5 0.350^*∗*^

^†^ANOVA post hoc; ^*∗*^Mann–Whitney; y, years; SD, standard deviation; F, female; UDVA, uncorrected distance visual acuity; CDVA, corrected distance visual acuity; UNVA, uncorrected near visual acuity; DCNVA, distance-corrected near visual acuity; SE, spherical equivalent; D, diopters; RE, right eye; LE, left eye.

**Table 2:** Characteristics of IOLs implanted in 160 patients who underwent cataract surgery.

Data	OptiVis	AR40e	M-Flex	ReZoom	ReSTOR
Manufacturer	Aaren Scientific	AMO	Rayner	AMO-Abbott	Alcon

Material	Hydrophilic acrylic, single piece	Hydrophobic acrylic with PMMA modified C haptic, three piece	Hydrophilic acrylic, single piece	Hydrophobic acrylic with PMMA modified C haptic, three piece	Hydrophobic acrylic, single piece

Optics	Hybrid (refractive and diffractive properties) multifocal, biconvex, aspheric	Monofocal, biconvex, aspheric	Refractive multifocal anterior surface, aspheric	Refractive multifocal anterior surface, aspheric	Diffractive multifocal

Near add spectacle plane	+2.80 D	0 D	+2.25 D	+2.50 D	+3.20 D

Light distribution	2 mm pupil diameter: 33% near, 38% intermediate, 27% far focus	100% far focus	2 mm pupil diameter: 18% near, 17% intermediate, 64% far focus	2 mm pupil diameter: 0% near, 17% intermediate, 80% far focus	2 mm pupil diameter: 38% near, 40% far focus
	5 mm pupil diameter: 20% near, 6% intermediate, 60% far focus		5 mm pupil diameter: 29% near, 10% intermediate, 60% far focus	5 mm pupil diameter: 30% near focus, 9% intermediate, 60% far focus	5 mm pupil diameter: 10% near, 84% far focus

Pupil dependence	Yes	No	Yes	Yes	Yes

Dimensions	Total diameter 11 mm; optic diameter 6 mm	Total diameter 13 mm; optic diameter 6 mm	Total diameter 12.5 mm; optic diameter 6.25 mm	Total diameter 13 mm; optic diameter 6 mm	Total diameter 13 mm; optic diameter 6 mm

Available powers	+10.00 D ÷ +30.00 D in 0.50 D increment	+10.00 D ÷ +30.00 D in 0.50 D increment	+14.00 D ÷ +25.00 D in 0.50 D increment	+6.00 D ÷ +30.00 D in 0.50 D increment	+10.00 D ÷ +30.00 D in 0.50 D increment

IOL, intraocular lens; mm, millimeter; D, diopter.

**Table 3:** Postoperative binocular visual acuity results at 3-month follow-up.

Parameter	Group 1 OptiVis	Group 2 (AR40e)	Group 3 M-Flex	Group 4 ReZoom	Group 5 ReSTOR	*p* value^*∗*^ between groups
Number of patients	32	32	32	32	32	
Number of eyes	64	64	64	64	64	

UDVA (logMAR)						1 versus 2 0.076
Mean ± SD	0.13 ± 0.12	0.08 ± 0.08	0.13 ± 0.11	0.09 ± 0.07	0.12 ± 0.10	1 versus 3 0.800
						1 versus 4 0.091
Range	0.52; 0.00	0.30; 0.00	0.40; 0.00	0.30; 0.00	0.40; 0.00	1 versus 5 0.864

CDVA (logMAR)						1 versus 2 0.094
Mean ± SD	0.07 ± 0.05	0.04 ± 0.05	0.09 ± 0.09	0.07 ± 0.06	0.08 ± 0.07	1 versus 3 0.198
						1 versus 4 0.989
Range	0.30; 0.00	0.15; 0.00	0.15; 0.00	0.30; 0.00	0.30; 0.00	1 versus 5 0.501

UNVA (logMAR)						1 versus 2 0.000
Mean ± SD	0.20 ± 0.14	0.43 ± 0.27	0.23 ± 0.16	0.17 ± 0.13	0.12 ± 0.13	1 versus 3 0.269
						1 versus 4 0.437
Range	0.52; 0.00	1.30; 0.00	0.70; 0.00	0.52; 0.00	0.40; 0.00	1 versus 5 0.009

DCNVA (logMAR)						1 versus 2 0.000
Mean ± SD	0.09 ± 0.06	0.43 ± 0.27	0.25 ± 0.17	0.18 ± 0.13	0.11 ± 0.13	1 versus 3 0.000
						1 versus 4 0.004
Range	0.22; 0.00	1.30; 0.00	0.70; 0.00	0.40; 0.00	0.40; 0.00	1 versus 5 0.824

SE (D) RE						1 versus 2 0.001
Mean ± SD	0.21 ± 0.59	−0.26 ± 0.49	−0.19 ± 0.39	−0.10 ± 0.28	0.04 ± 0.47	1 versus 3 0.002
						1 versus 4 0.011
Range	−1.50; 1.00	−1.75; 0.75	−1.00; 0.75	−1.00; 0.25	−1.50; 1.00	1 versus 5 0.296

SE (D) LE						1 versus 2 0.005
Mean ± SD	0.14 ± 0.65	−0.28 ± 0.58	−0.13 ± 0.39	−0.08 ± 0.31	0.20 ± 0.50	1 versus 3 0.026
						1 versus 4 0.054
Range	−1.25; 2.00	−2.75; 0.5	−1.00; 0.75	−1.00; −0.50	−1.25; 1.25	1 versus 5 0.420

^*∗*^Mann–Whitney; SD, standard deviation; UDVA, uncorrected distance visual acuity; CDVA, corrected distance visual acuity; UNVA, uncorrected near visual acuity; DCNVA, distance-corrected near visual acuity; SE, spherical equivalent; D, diopter; RE, right eye; LE, left eye.

**Table 4:** Mesopic log contrast sensitivity function at far distances without glare in multifocal intraocular lenses at 3-month follow-up.

Parameter	Group 1 OptiVis	Group 3 M-flex	Group 4 ReZoom	Group 5 ReSTOR	*p* value^*∗*^
Number of patients	32	32	32	32	
Number of eyes	64	64	64	64	

CS at 1.5 cpd					1 versus 3 0.288
Mean ± SD	1.56 ± 0.25	1.43 ± 0.22	1.42 ± 0.21	1.58 ± 0.22	1 versus 4 0.222
					1 versus 5 0.996

CS at 3 cpd					1 versus 3 0.560
Mean ± SD	1.68 ± 0.38	1.57 ± 0.25	1.68 ± 0.21	1.72 ± 0.19	1 versus 4 1.000
					1 versus 5 0.986

CS at 6 cpd					1 versus 3 0.968
Mean ± SD	1.26 ± 0.66	1.36 ± 0.56	1.56 ± 0.49	1.50 ± 0.38	1 versus 4 0.293
					1 versus 5 0.503

CS at 12 cpd					1 versus 3 0.909
Mean ± SD	0.38 ± 0.54	0.53 ± 0.55	0.66 ± 0.68	0.45 ± 0.57	1 versus 4 0.482
					1 versus 5 0.994

CS at 18 cpd					1 versus 3 0.986
Mean ± SD	0.19 ± 0.34	0.13 ± 0.29	0.48 ± 0.49	0.14 ± 0.34	1 versus 4 0.240
					1 versus 5 0.994

^*∗*^ANOVA post hoc; CS, contrast sensitivity; spatial frequencies of 1.5, 3, 6, 12, and 18 cycles per degree; SD, standard deviation.

**Table 5:** Visual function in multifocal IOLs at 3-month follow-up.

Parameter	Group 1 OptiVis	Group 3 M-Flex	Group 4 ReZoom	Group 5 ReSTOR	*p* value^*∗*^
Number of patients	32	32	32	32	
Number of eyes	64	64	64	64	

Spectacle dependence (far)	16	3	0	6	1 versus 3 0.089
					1 versus 4 0.021
					1 versus 5 0.223

Spectacle dependence (near)	50	44	44	16	1 versus 3 0.619
					1 versus 4 0.619
					1 versus 5 0.004

Presence of dysphotopsia (spontaneously mentioned)	34	25	41	25	1 versus 3 0.415
					1 versus 4 0.608
					1 versus 5 0.415

Presence of dysphotopsia (by questionnaire)	59	53	59	38	1 versus 3 0.617
					1 versus 4 1.000
					1 versus 5 0.082

Visual function					1 versus 3 0.286
Mean ± SD	89.28 ± 11.11	84.62 ± 13.91	87.73 ± 11.19	89.51 ± 14.85	1 versus 4 0.686
Range	50.00; 100.00	55.36; 100.00	58.93; 100.00	37.50; 100.00	1 versus 5 0.300

^*∗*^Mann–Whitney; SD, standard deviation.
